# Hypothermic Oxygenated Machine Perfusion (HOPE) During Total Vascular Exclusion With Veno‐Venous Bypass for Giant Hepatic Hemangioma Resection

**DOI:** 10.1111/aor.15065

**Published:** 2025-08-05

**Authors:** Mehdi Boubaddi, Florence Jeune, Chetana Lim, Claire Goumard, Eric Savier, Géraldine Rousseau, Fabiano Perdigao, Olivier Scatton

**Affiliations:** ^1^ Department of Digestive, Hepato‐Biliary and Pancreatic Surgery and Liver Transplantation AP‐HP Pitié‐Salpêtrière Hospital Paris France; ^2^ Sorbonne Université Paris France; ^3^ Research Unit Université de Picardie‐Jules Verne, UR UPJV 7518 SSPC Amiens France; ^4^ Centre de Recherche de Saint‐Antoine (CRSA), INSERM, UMRS‐938 Paris France

**Keywords:** hypothermic oxygenated machine perfusion (HOPE), in situ‐HOPE, major liver resection, post hepatectomy liver failure (PHLF), post operative ischemia‐reperfusion lesion, total vascular exclusion (TVE), veno‐venous bypass

## Abstract

Hypothermic oxygenated machine perfusion (HOPE) during total vascular exclusion with veno‐venous bypass for major hepatic resection is a safe procedure and could help to reduce ischemia–reperfusion lesions and related complications.

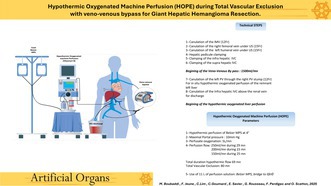

## 1 Introduction

Total vascular exclusion (TVE) may be required to safely resect tumors involving the inferior vena cava (IVC) or hepatocaval confluence and prevent intra‐operative hemorrhage and air embolism [1, 2, 3]. When a TVE > 60 min is planned, veno‐venous bypass associated with hypothermic portal perfusion is recommended to reduce warm ischemia and posthepatectomy liver failure (PHLF).

Hypothermic oxygenated machine perfusion (HOPE) has demonstrated protective effects against ischemia–reperfusion injury and biliary damage in liver transplantation (LT) compared to static cold storage. The potential benefit of HOPE in the setting of extended liver resection with prolonged ischemia has yet to be widely explored.

We present a case of benign tumor resection under prolonged TVE with veno‐venous bypass using HOPE.

## 2 Case Presentation

A 56‐year‐old woman presented with a history of abdominal mass syndrome with altered general condition, anorexia, and weight loss.

**FIGURE 1:**
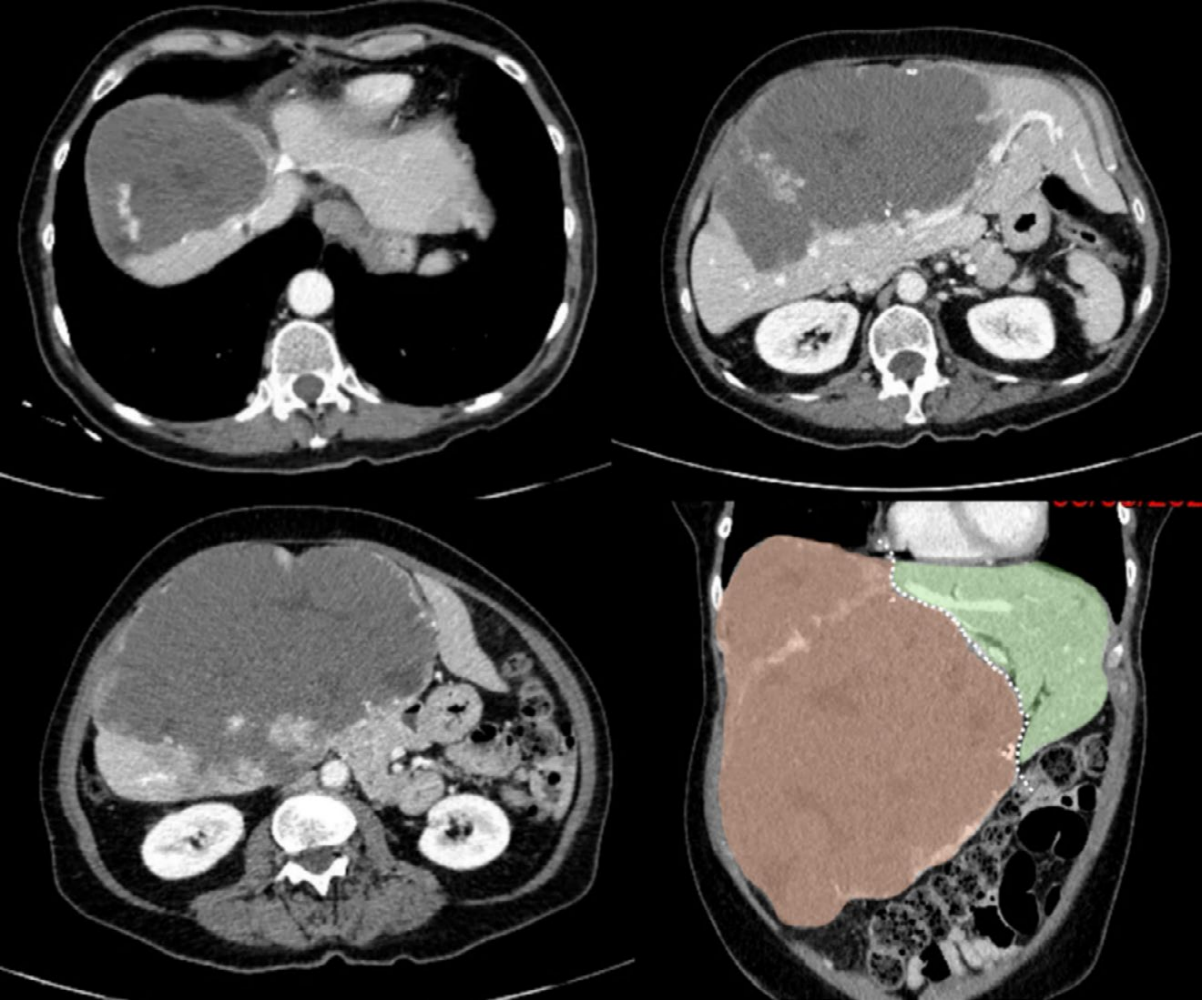
CT scan with giant hemangioma in the right liver. [Color figure can be viewed at wileyonlinelibrary.com]

Biological tests revealed cholestasis and thrombopenia related to Kasabach–Meritt syndrome. The contrast‐enhanced scan demonstrated a giant hemangioma of the right liver compressing the right and middle hepatic veins as well as the left bile duct (Figure 1).

Liver volumetry showed a total hepatic volume of 4026 mL, with the right lobe (S4‐5‐6‐7‐8) contributing 3420 mL. The tumor‐free right lobe was estimated at 626 mL (53%) while the tumor‐free left lateral lobe (S2‐3) measured 560 mL (47%).

After failed embolization with bleomycin, we recommended during a multidisciplinary meeting to perform liver surgery under TEV with intraoperative veno‐venous bypass and HOPE perfusion as an alternative to LT.

### 2.1 Surgical Steps and Machine Parameters

**FIGURE 2:**
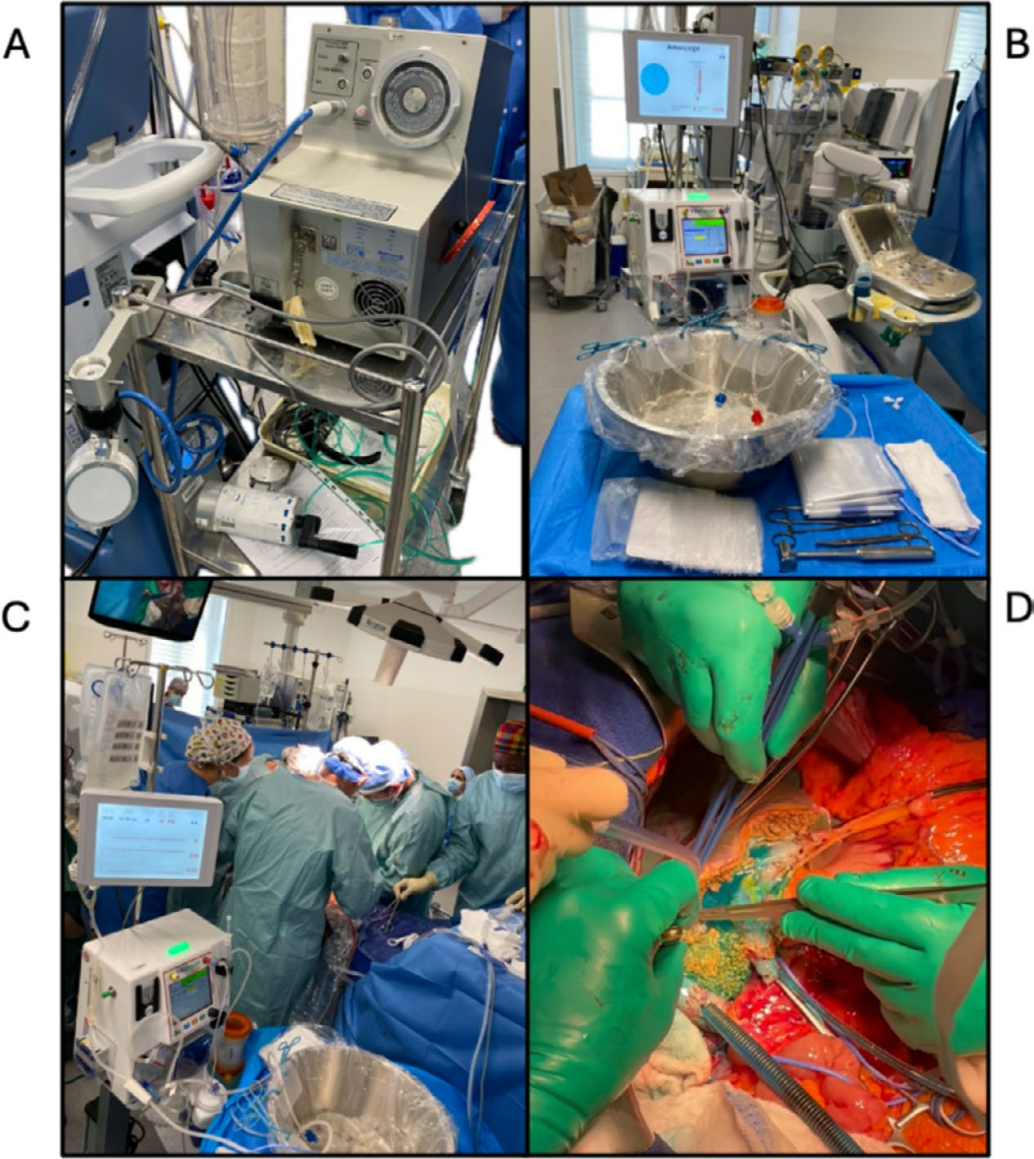
Veno‐venous bypass (A) with in situ hypothermic perfusion under total vascular exclusion (B) with hypothermic oxygenated machine perfusion (bridge to life) (C). Blue rewarming at the end of liver resection for bile leakage and flush out the conservation liquid before declamping. [Color figure can be viewed at wileyonlinelibrary.com]

**FIGURE 3:**
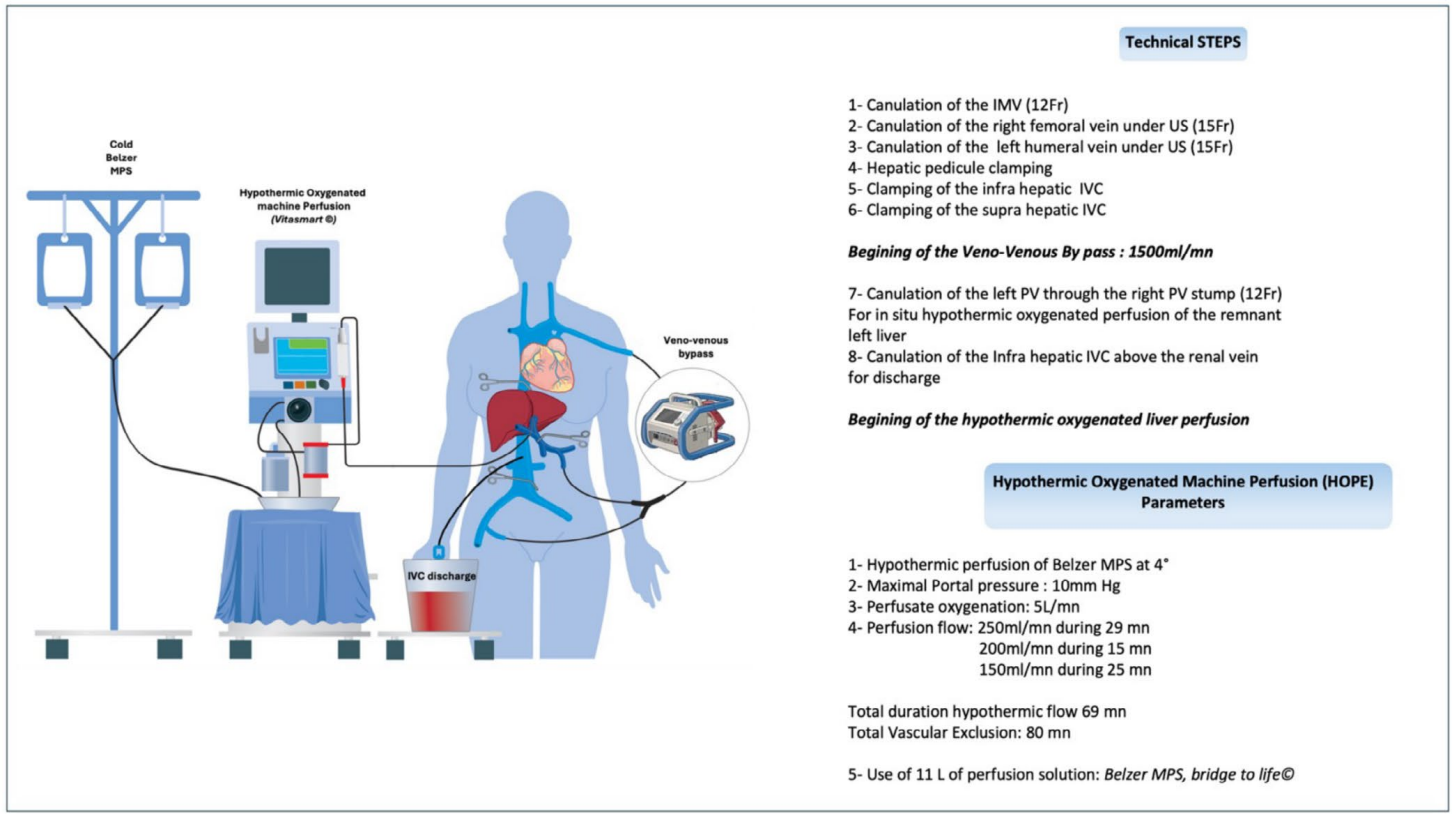
Surgical setup with patient, hypothermic oxygenated machine perfusion (bridge to life), and veno‐venous bypass. IVC, inferior vena cava; IVM, inferior mesenteric vein; MPS, machine perfusion solution; PV, portal vein. [Color figure can be viewed at wileyonlinelibrary.com]

The surgical setup is illustrated in Figures 2 and 3.

First, a complete mobilization of the liver and vascular control of the infra and suprahepatic inferior vena cava was performed.

After ligation of the right portal vein (rPV) and right hepatic artery (rHA), TVE and percutaneous veno‐venous bypass were initiated.

The HOPE setup included a perfusate of two liters Belzer MPS solution cooled to 4°C, connected to the rPV stump to preserve the left portal branch via a 14 Fr inflow cannula.

Outflow was provided through a 30 Fr cannula placed in the IVC to drain the oxygenated perfusate.

The portal inflow cannula targeted a maximum portal pressure of 10 mmHg. Oxygenated perfusate flow was monitored at 5 L/min, targeting a 60–80 kPA pressure. The preservation solution was maintained between 4°C and 6°C.

A right lobectomy was performed. The middle hepatic vein (HV) was divided near the tumor and the right HV at the end of the transection using a vascular stapler. During resection, the perfusion flow rate was adjusted to ensure a maximal PV pressure of 10 mmHg, as follows: 250 mL/min (29 min), 200 mL/min (15 min), 150 mL/min (25 min). HOPE lasted 69 min and total TVE duration was 80 min.

After tumor resection, gradual rewarming and sequential declamping were performed. A methylene blue test confirmed hemostasis and biliostasis. An internal biliary drain was placed, and Doppler ultrasound confirmed vascular patency. Estimated blood loss was < 200 mL. No transfusion was required.

The postoperative course was uneventful with no PHLF (Balzan's 50–50 criteria) [4]. Liver tests were normalized at postoperative day (POD) 5. The patient was discharged at POD 7.

Pathological examination confirmed complete resection (R0) of a 24 cm cavernous hemangioma.

## 3 Discussion

This report describes, to our knowledge, the first case of right lobectomy performed under TVE, and veno‐venous bypass combined with HOPE. The procedure was well‐tolerated with no postoperative complications, especially no PHLF.

Hypothermic perfusion has been previously proposed to protect the FLR by reducing metabolic demand and decreasing ischemic injury during TVE.

In 2015, Azoulay et al. [5] published a series of 77 complex liver resections performed under total vascular exclusion (TVE) with hypothermic portal perfusion and veno‐venous bypass. Their technique involved in situ cooling using nonoxygenated static preservation solution. The series included mostly malignant tumors, with interesting results in highly selected patients despite a high 90‐day mortality (19.5%) exclusively related to PHLF.

HOPE, first developed for LT, offers an additional layer of protection by oxygenating the parenchyma and promoting mitochondrial function prior to reperfusion. Recent reports suggest that HOPE, in addition to cooling during complex liver resection, may reduce ischemia–reperfusion injury, decrease PHLF, and improve early postoperative outcomes.

Cillo et al. [6], have reported a series of five patients who underwent an ante situ liver resection using HOPE for intrahepatic cholangiocarcinomas. This technique involved extensive mobilization of the liver with caval and hepatic vein reconstruction. No postoperative liver failure or deaths were encountered, and liver function tests were normalized within the first week.

Recently, Tribolet et al. [7] reported a case of hepatic resection involving the cavo‐hepatic confluence using TVE and HOPE, with preserved caval flow through a porto‐caval anastomosis. When feasible, preservation of caval flow by clamping only the hepatic veins avoids veno‐venous bypass complications but requires ligation of all the retrohepatic veins to prevent any passage of cold preservation solution (4°C) into the systemic circulation with a risk of cardioplegia.

In contrast with the previous cases [5, 6], our approach provides active temperature, flow, and oxygenation control throughout the procedure without the risk of cardioplegia. We used HOPE in an in situ resection setting for a benign tumor with no need for vascular reconstruction or liver mobilization outside of the abdominal cavity.

## 4 Conclusion

HOPE may reduce the risk of ischemia–reperfusion injury and related complications, especially for complex liver resection requiring prolonged TVE. Further prospective studies could compare postoperative outcomes between HOPE and standard hypothermic perfusion in this setting.

## Author Contributions

**Olivier Scatton:** design, perform the surgery, writing and review. **Mehdi Boubaddi:** writing the paper, editing. **Florence Jeune:** co writing, editing and critical review. **Fabiano Perdigao:** perfom surgery, critical review. **Géraldine Rousseau:** critical review. **Claire Goumard:** critical review, design the paper. **Chetana Lim:** editing, critical review. **Eric Savier:** critical reveiw and editing.

## Conflicts of Interest

The authors declare no conflicts of interest.
